# Female Malignancies and Immunotherapy: What’s New?

**DOI:** 10.3390/cancers12102909

**Published:** 2020-10-10

**Authors:** Silvia Pesce, Valerio Gaetano Vellone, Emanuela Marcenaro

**Affiliations:** 1 Department of Experimental Medicine (DI.ME.S.) and Centre of Excellence for Biomedical Research (CEBR), University of Genoa, 16132 Genoa, Italy; 2 Department of Integrated Surgical and Diagnostic Sciences (DISC), University of Genoa, 16132 Genoa, Italy; 3 Anatomic Pathology University Unit, IRCCS Ospedale Policlinico San Martino, 16132 Genoa, Italy

For many years, the therapeutic advances in gynecological neoplasms have remained steady, however, in recent years, the application of the most modern “-omics” sciences has shed light on the pathogenesis and on neoplastic progression, with important implications in the introduction of targeted treatments that are more effective and less toxic [1,2]. 

Current emerging therapies for solid tumors include various immunotherapeutic approaches, like immune checkpoint inhibitors (ICIs) [3,4] that have gained considerable attention because of their impressive treatment outcomes in different tumor types. Unfortunately, benefits have been seen only in a small percentage of patients with solid tumors. For this reason, it is strictly necessary to implement our knowledge on immune checkpoints (ICs) and on the tumor microenvironment. 

In the cancer treatment context, T cells have always been considered to be primarily responsible for the beneficial effect of immunotherapy. In addition to T cells, natural killer (NK) cells are also now seen as a promising cancer immunotherapy tool due to their ability to kill malignant cells without toxicity towards healthy cells [5,6].

Unfortunately, the function of these effector cell types is often drastically altered in the tumor microenvironment and this event can contribute to tumor progression and metastasis.

Among existing cancers, female malignancies represent one of bugbears in terms of diagnosis and clinical approach efficacy. Indeed, female tumors are often characterized by an immunosuppressive tumor environment able to resist not just immune system attack, but also classical anticancer drugs [7,8]. For this reason, immunotherapy represents the most promising therapeutic approach in gynecological cancer. 

Among the most promising approaches to activate therapeutic antitumour immunity is the blockade of programmed cell death protein 1 (PD-1) [9,10]. PD-1 was first discovered on T cells, where it helps keep T cells from attacking other cells in the body. Drugs blocking PD-1 boost the immune response against cancer cells. This can shrink some tumors or slow their growth. Recently, the PD-1 immune checkpoint has also been identified on NK cells, revealing a possible role for this receptor blockade in restoring the antitumor activity of these potent cytotoxic cells too. Interestingly, it has been shown that the ovarian tumor microenvironment is enriched in PD-1+ NK cells [11].

In the gynecological context, current and ongoing studies are trying to improve clinical responses through immunotherapy strategies combined or not with classic treatments. Indeed, atezolizumab can be used with Abraxane (albumin-bound paclitaxel) for advanced triple-negative breast cancer when the tumor makes the PD-L1 protein [12]. It can even be used as part of the first treatment in some people. Pembrolizumab can be used by itself in women with endometrial cancers that are mismatch repair deficient (dMMR) or microsatellite instability high (MSI-H). Both of these are linked to high numbers of gene mutations (changes) in the cancer cells. Tumors can be tested for these uncommon changes.

Pembrolizumab can also be used along with the targeted drug lenvatinib (Lenvima) in women with advanced endometrial cancers that are not MMR deficient (dMMR) or microsatellite instability high (MSI-H), typically after at least one other drug treatment has been tried. In addition, immunotherapy makes sense in cervical cancer—a virally induced tumor that should be highly angiogenic. Virus-induced cancers generally make good targets for immunotherapy because viral proteins are strong immune stimulants. Based on these preliminary data, the FDA granted an accelerated approval in June 2018 to pembrolizumab for the treatment of patients with advanced cervical cancer who have disease progression during or after chemotherapy, whose tumors express PD-L1 (combined positive score ≥ 1).

In light of this, the scope of this Special Issue is to contribute to dissecting molecular and cellular immunological mechanisms involved in female malignancy progression and metastasis and to take stock of immunotherapy successes and failures in these tumors. This information will provide new insights crucial for the development of new immunotherapeutic strategies and for the improvement of combination strategies in patient-specific female cancer treatments.
